# Pre-thrombectomy prognostic prediction of large-vessel ischemic stroke using machine learning: A systematic review and meta-analysis

**DOI:** 10.3389/fneur.2022.945813

**Published:** 2022-09-08

**Authors:** Minyan Zeng, Lauren Oakden-Rayner, Alix Bird, Luke Smith, Zimu Wu, Rebecca Scroop, Timothy Kleinig, Jim Jannes, Mark Jenkinson, Lyle J. Palmer

**Affiliations:** ^1^Australian Institute for Machine Learning, University of Adelaide, Adelaide, SA, Australia; ^2^School of Public Health, University of Adelaide, Adelaide, SA, Australia; ^3^Department of Radiology, Royal Adelaide Hospital, Adelaide, SA, Australia; ^4^School of Public Health and Preventive Medicine, Monash University, Melbourne, VIC, Australia; ^5^Faculty Health and Medical Science, School of Medicine, University of Adelaide, Adelaide, SA, Australia; ^6^Department of Neurology, Royal Adelaide Hospital, Adelaide, SA, Australia; ^7^Functional Magnetic Resonance Imaging of the Brain Centre, University of Oxford, Oxford, United Kingdom

**Keywords:** ischemic stroke, large vessel occlusion, endovascular thrombectomy, prognostic prediction, machine learning, deep learning

## Abstract

**Introduction:**

Machine learning (ML) methods are being increasingly applied to prognostic prediction for stroke patients with large vessel occlusion (LVO) treated with endovascular thrombectomy. This systematic review aims to summarize ML-based pre-thrombectomy prognostic models for LVO stroke and identify key research gaps.

**Methods:**

Literature searches were performed in Embase, PubMed, Web of Science, and Scopus. Meta-analyses of the area under the receiver operating characteristic curves (AUCs) of ML models were conducted to synthesize model performance.

**Results:**

Sixteen studies describing 19 models were eligible. The predicted outcomes include functional outcome at 90 days, successful reperfusion, and hemorrhagic transformation. Functional outcome was analyzed by 10 conventional ML models (pooled AUC=0.81, 95% confidence interval [CI]: 0.77–0.85, AUC range: 0.68–0.93) and four deep learning (DL) models (pooled AUC=0.75, 95% CI: 0.70–0.81, AUC range: 0.71–0.81). Successful reperfusion was analyzed by three conventional ML models (pooled AUC=0.72, 95% CI: 0.56–0.88, AUC range: 0.55–0.88) and one DL model (AUC=0.65, 95% CI: 0.62–0.68).

**Conclusions:**

Conventional ML and DL models have shown variable performance in predicting post-treatment outcomes of LVO without generally demonstrating superiority compared to existing prognostic scores. Most models were developed using small datasets, lacked solid external validation, and at high risk of potential bias. There is considerable scope to improve study design and model performance. The application of ML and DL methods to improve the prediction of prognosis in LVO stroke, while promising, remains nascent.

**Systematic review registration:**

https://www.crd.york.ac.uk/prospero/display_record.php?ID=CRD42021266524, identifier CRD42021266524

## Introduction

Ischemic stroke caused by large vessel occlusion (LVO) accounts for 24–46% of ischemic stroke cases (1). Endovascular thrombectomy (EVT) is currently the standard care for ischemic stroke patients with occlusion in the anterior cerebral circulation and salvageable brain tissue within 24 h of symptom onset (2). However, despite advances in stroke treatment, the rate of long-term disability/dependency is up to approximately 50% in LVO patients (3). Further, EVT is resource intensive. Better identification of the risks and benefits of intervention may be valuable to optimize patient outcomes and reduce healthcare and societal costs.

To help improve treatment strategies and clinical decision-making, prior studies have investigated pre-treatment predictors of key clinical outcomes following LVO stroke, including comorbidities, clinical examination, and neuroimaging findings (4). A number of prognostic scores using simple linear combinations of these predictors, such as ASPECTS, HIAT, and MR PREDICTS, have been constructed and validated in LVO cohorts treated with EVT (4). However, they may have low clinical utility due to their modest performance in practice (4). Other barriers of their clinical implementation include complexity of scoring and the subjective nature of data acquisition, which are time-dependent with concomitant high inter-observer variability (5, 6). There is a need for a more robust and clinically useful prognostic tool.

Machine learning (ML) techniques are being increasingly applied to clinical tasks (7). These techniques have the potential to handle a large quantity of data and identify latent patterns and complex relationships (8). Deep learning (DL), a newer type of ML technique, can automatically learn useful features at the pixel or voxel level, which is particularly powerful in processing raw medical images (9). DL has shown substantial promise in clinical prognostic prediction based on raw image data (10, 11), and, therefore, may play a role in predicting stroke outcomes—an area characterized by rich neuroimaging datasets.

This systematic review aimed to evaluate the performance, validity, and clinical applicability of published ML-based pre-thrombectomy prognostic models for LVO stroke and to identify key research gaps.

## Methods

This systematic review was registered on PROSPERO (12) (ID: CRD42021266524) and conducted in line with the PRISMA guidelines (13).

### Eligibility criteria

Publications were eligible for inclusion if the study applied ML and/or DL algorithms to predict clinical outcomes following EVT treatment of LVO stroke. Specifically, the studies were included if: 1) the prediction models were applied to LVO stroke patients treated with EVT; and 2) the study employed ML-based algorithms, such as random forest analysis, naive Bayes classifiers, support vector machines, regression models, and/or various DL algorithms such as convolutional neural networks. Standard regression models without penalization (such as simple logistic regression, linear regression, and cox regression models) were not considered within the scope of this review.

Studies were excluded if: 1) the prediction models included patients with non-LVO stroke such as intracerebral hemorrhage or lacunar stroke; 2) assessment of the model performance was not performed; or 3) the prediction models involved post-EVT information. Conference abstracts, review articles, letters, comments, editorials, and erratum were excluded due to limited information contained.

### Search strategies

Full details of the search strategies are shown in Supplementary Table S1. A variety of keywords were selected for literature search after consultation with an academic librarian. Systematic searches were conducted in four databases—PubMed, Embase, Scopus, and Web of Science, from inception until the 18th February 2022. These databases included related computer science conferences and journal papers, except the International Conference on Medical Imaging with Deep Learning (MIDL), so manual searches in MIDL were conducted to supplement the searches in online databases. Searches were limited to studies published in English.

### Study selection

Two reviewers (MZ and ZW) independently conducted study selection and review. After removing duplicates, conference abstracts, narrative reviews, comments, letters, editorial and erratum, the records were screened based on the titles and abstracts, and subsequently assessed by full-text reading. Discrepancies between the two reviewers were resolved by discussion and consultation with a third reviewer (LJP).

### Data extraction

Relevant data from the eligible studies were extracted into a pre-specified form independently by two reviewers (MZ and ZW). The data extracted were: 1) year of publication; 2) sample sizes of the training, testing, and external validation cohorts if applicable; 3) demographic characteristics of the study population (age, gender, and ethnicity/place of recruitment); 4) vessel occlusion sites; 5) clinical outcomes assessed; 6) imaging modality used for model development; 7) specific algorithms used; 8) model performance; and 9) model validation. Information related to model development and model performance was restricted to that pertaining to the “best-performing” model. A third reviewer (LJP) resolved any disagreements regarding the extracted information between the two reviewers.

### Data synthesis

The model performance was quantified by area under the receiver operating characteristic curve (AUC), an estimation for the discriminative capacity of a model. The AUCs and 95% confidence intervals (CIs) of relevant models were extracted and synthesized. The standard error of each AUC was calculated using the actual positive endpoint and actual negative endpoint based on formula provided in Bradley et al. (14). To make analyses consistent, 95% CIs were calculated based on the information available in the reports using the statistical formula (15): 95% CI = effect size (AUC) ± 1.96 × standard error. “Significant” statistical heterogeneity was defined using the Cochran's *Q*-test (*P* ≤ 0.10) and the I^2^ statistic (>50%) (16). AUCs were pooled in a random-effects model if there was significant heterogeneity suggested by the *Q*-test or I^2^. Otherwise, the AUCs were pooled using a fixed-effects model. For adequate statistical power, we used Egger's test with a funnel plot to detect publication bias only when a meta-analysis included more than 10 AUCs and had no statistically substantial heterogeneity suggested by the I^2^ or *Q*-test (17, 18). The meta-analyses were conducted using the MedCalc Statistical Software (version 20.0.3).

### Risk of bias and reporting quality

Assessment of risk of bias was conducted using the Prediction Model Risk of Bias Assessment Tool (PROBAST) (19). This tool contains 20 questions covering four domains, including participants, predictors, outcomes, and analysis. Assessment of the adherence to reporting standards was conducted using the Transparent Reporting of a Multivariable Prediction Model for Individual Prognosis Or Diagnosis (TRIPOD) protocol (20). This checklist contains 22 items (37 points) covering multiple aspects, including title and abstract, backgrounds and objectives, methods, results, discussion, supplementary and funding. In TRIPOD and PROBAST, items related to the details of predictors were not applicable for studies using DL models. This was because “predictors” in DL models are usually each pixel or voxel of an image, which are less likely to be reported in DL models (21). The modified TRIPOD and PROBAST are shown in Supplementary Tables S2, S3.

## Results

### Search results

**Figure 1:**
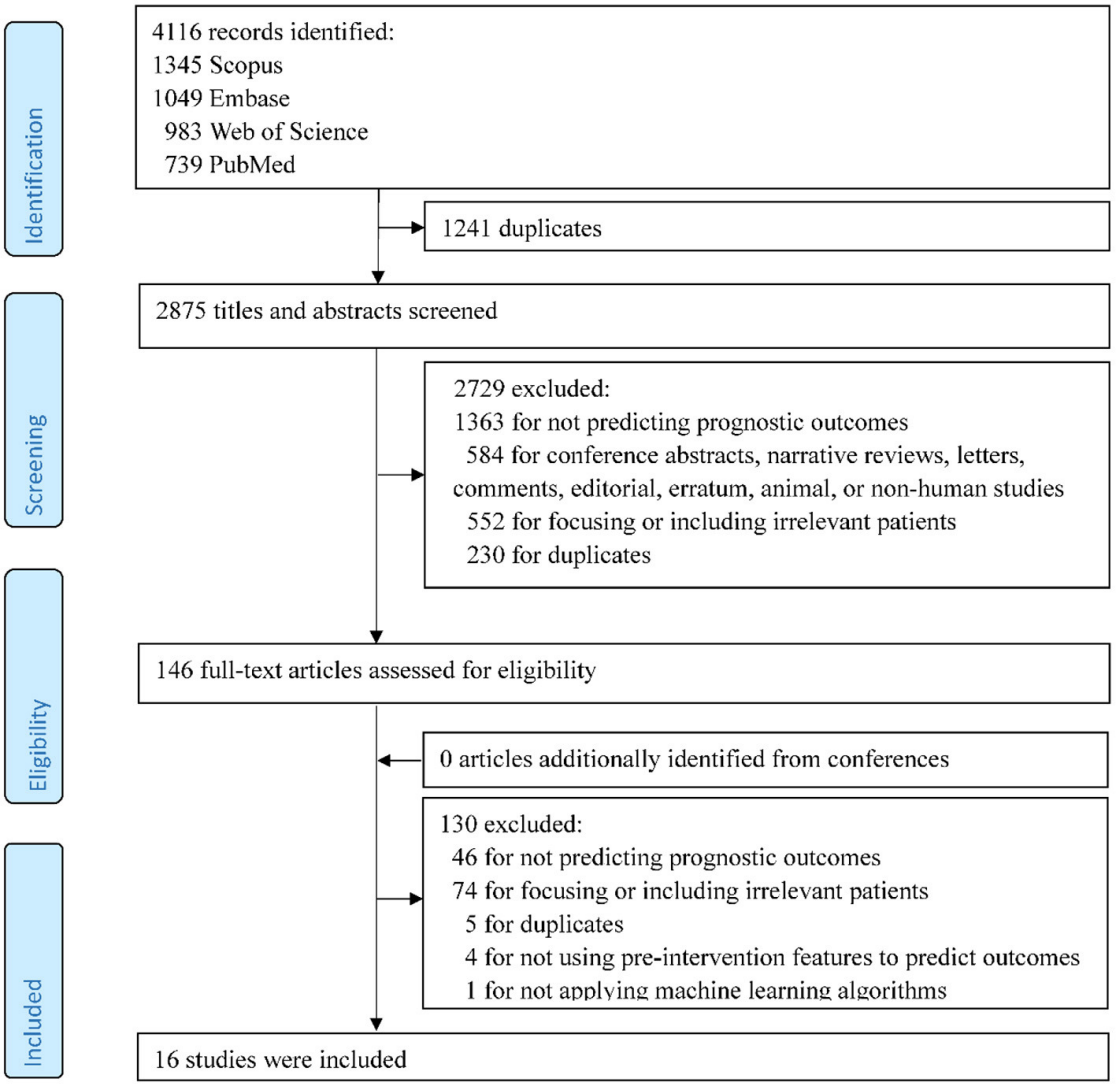
Flow chart of study selection.

A total of 4,116 records were identified in the initial search. After the review of titles and abstracts and the screening of full texts, 16 studies met the inclusion criteria and were included in the systematic review (Figure 1).

### Basic characteristics

The basic characteristics of the eligible studies (22–37) are summarized in Supplementary Table S4. The mean or median ages of the study participants ranged from 64.0 to 86.0 years, and the proportion of male participants ranged from 35.0 to 65.9%. Only one US study (24) specifically described the self-reported ethnicity of the patients (63.0–69.0% European ancestry); the other studies reported the place of patient recruitment [USA: 1 (32); Europe: 10 (22, 23, 26–29, 31, 33–35); Asia: 4 (25, 30, 36, 37)]. The training sample sizes ranged widely, from 109 to 1,401. Regarding the testing sample, two studies used hold-out test sets, respectively containing 208 patients (30) and 100 patients (35). The remaining studies performed cross-validation (23–26, 28, 29, 31–34, 36, 37) or bootstrap approach (22, 27). The five studies (23, 29, 31, 34, 35) used data obtained from MR CLEAN Registry (38). Fifteen studies reported the occlusion sites, of which 14 studies (22, 23, 25–36) included patients with anterior circulation occlusion and one (24) further included patients with occlusion in the posterior circulation.

### Model development

#### Conventional machine learning algorithms

Details of model development in 12 studies using conventional ML algorithms are shown in Table 1. Tree models (22, 24, 31), random forests (23, 26, 27), and support vector machines (28, 30, 33) were each proposed by three studies, regularized logistic regression by two studies (25, 32), and artificial neural networks by one study (29). To accommodate missing values, two studies used multiple imputation (23, 29) and one used singular imputation (31), while other studies excluded participants with missing data in either predictive or outcome variables (complete-case analysis) (22, 24–28, 30, 32, 33). The number of predictive variables used for model construction varied from 4 (32) to 53 (23). The National Institutes of Health Stroke Scale and age were commonly ranked as the important predictors. All studies conducted internal validation, either by bootstrapping (22, 27), hold-out validation (30), or k-fold cross-validation (23–26, 28, 29, 31–33).

**Table 1:** Model development using conventional machine learning algorithms.

**References**	**Model**	**Outcomes**	**Missing value**	**Features**	**Important feature identified**	**Validation**
Brugnara et al. (22)	Tree model: Gradient boosting decision trees	Good functional outcomes (mRS ≤ 2)	Patients with missing data were excluded	16	Premorbid mRS, baseline acute ischemic volume, NIHSS, onset to imaging time, baseline eASPECTS	Bootstrapping (25 bootstrap sample)
Van et al. (23)	RFA	a. Good functional outcomes (mRS ≤ 2) b. Successful reperfusion (TICI score≥2b)	Patients with missing data of main outcomes were excluded; other variables, multiple imputations	53	Age, NIHSS at baseline, duration of onset to groin puncture, Glasgow Coma Scale, systolic BP at baseline, CRP, creatinine, thrombocyte count, diastolic BP at baseline, baseline ASPECTS, glucose, clot burden score; feature importance for good functional outcomes only: baseline mRS, presence of leukoaraiosis, collateral score; feature importance for successful reperfusion only: occlusion site, hyperdense artery sign, history of AF	Nested cross-validation: 100 repeated random splits; 10-fold cross validation
Alawieh et al. (24)	Tree model (regression tree)	Good functional outcomes (mRS ≤ 2) ^*^	Patients with missing data were excluded	12	Age, gender, race, diabetes, hypertension, hyperlipidemia, arterial fibrillation, preceding intravenous thrombolysis, onset to groin puncture time, NIHSS, baseline mRS, ASPECTS^**^	10-fold cross-validation
Nishi et al. (25)	RLR	Good functional outcomes (mRS ≤ 2)	Patients with missing data were excluded	16	Care-dependent, age, premorbid mRS, ASPECTS, NIHSS	10-fold cross-validation
Hamann et al. (26)	RFA	Good functional outcomes (mRS ≤ 2)	Patients with missing data were excluded	10	Age, NIHSS at baseline, systolic blood pressure, risk factors (hypertension, diabetes, smoking, previous ischemic event), preceding intravenous thrombolysis, onset to groin puncture time, collateralization status, perfusion value of the medial MCA territory, volume of core, and volume of tissue at risk^**^	5-fold cross validation
Kerleroux et al. (27)	RFA	Good functional outcomes (mRS ≤ 3)	Patients with missing data were excluded	32	Receiving mechanical thrombectomy, the absence of ICA occlusion, lower HE-I, decreasing age, and the presence of eloquent mismatch within the following regions: the right thalamus, the left thalamus, the left superior longitudinal fasciculus, the left post central gyrus, the left retro-lenticular part of internal capsule, and the left supra marginal gyrus	Bootstrapping
Xie et al. (28)	SVM	Good functional outcomes (mRS ≤ 2)	Patients with missing data were excluded	4	Age, baseline NIHSS score, lesion volume, ischemic percentage in each brain region	Nested cross-validation: 100 repeated random splits; 10-fold cross validation
Ramos et al. (29)	ANN	Poor functional outcomes (mRS≥5)	Multiple imputation	51	Age, collateral, glucose level, NIHSS, and pre-stroke mRS	Nested cross-validation: 10 equally sized splits; 5-fold cross validation
Ryu et al. (30)	SVM	Poor functional outcome (mRS≥4)	Patients with missing data were excluded	6	Age, NIHSS, hypertension, diabetes mellitus, AF, and poor collateral^*^	Hold-out validation
Kappelhof et al. (31)	Tree model (Decision tree)	Poor functional outcome (mRS≥5)	Singular imputation	6	Age, pre-stroke mRS, start of endovascular thrombectomy, NIHSS at baseline, history of diabetes mellitus, duration of CTA in first hospital to groin puncture^*^	5-fold cross-validation
Patel et al. (32)	RLR	Successful reperfusion at the first attempt (TICI score≥2b)	Patients with missing data were excluded	4	Clot length, clot perviousness, distance from internal carotid artery, angle between the aspiration catheter and the clot	Nested cross-validation: 100 repeated random splits; 10-fold cross validation
Hofmeister et al. (33)	SVM	Successful reperfusion at the first attempt (TICI score≥2b)	Patients with missing data were excluded	9	Large area low gray level emphasis, gray level variance, large dependence emphasis, short run emphasis, entropy, maximum, run percentage, coarseness, and gray level nonuniformity normalized^*^	10-fold cross-validation

AF, atrial fibrillation; ASPECTS, The Alberta stroke program early CT score; BP, blood pressure; CRP, C-reactive protein; HE-I, high-eloquence infarct; MCA, middle cerebral artery; mRS, Modified Rankin Scale; ICA, internal carotid artery; NIHSS, The National Institutes of Health Stroke Scale; RFA, random forest analysis; RLR, regularized logistic regression; TICI, thrombolysis in cerebral infarction score; n.a., not available; ANN, artificial neural networks; CTA, computed tomography angiography; mRS, Modified Rankin Scale; SVM, support vector machine; TICI, thrombolysis in cerebral infarction score.

* Study used regression tree model to predict continuous multiclass mRS (0, 1, 2, 3, 4, 5, 6) and also dichotomized multiclass mRS (“good” vs. “poor” function) for model prediction and comparison.

** Features used in final model were listed here as feature importance ranking analysis was not conducted in the included study.

#### Deep learning algorithms

Table 2 summarizes the model development of DL algorithms in four studies. All studies conducted skull stripping, augmentation, normalization, and imaging resampling (34–37). Two studies (36, 37) additionally labeled regions of interest in the scans. All studies used DL algorithms based on supervised learning (34–37), with one study also using unsupervised learning (auto-encoder) for model pre-training (34). Regarding model architectures, Hilbert et al. (34) used a convolutional auto-encoder to obtain representative imaging features and applied a 2-D ResNet for fine-tuning in successful reperfusion prediction, while the auto-encoder was not used in the best model for functional outcome prediction. The authors utilized structured receptive field kernels (as opposed to learned convolutional kernels) to help prevent overfitting. Samak et al. (35) and Jiang et al. (37) both used a 3-D CNN feature encoder and incorporated imaging and clinical data using metadata fusion technique. The former additionally used self-attention technique (squeeze and excitation modules) in their encoders, while the latter is based on pre-trained Inception V3 encoders. Additionally, the latter built the encoder individually on multiple imaging modalities (Diffusion Weight Imaging [DWI], Mean Transit Time map, and Time To Peak map). Nishi et al. (36) used a U-net for predicting ischemic core lesion segmentation to derive feature representations and used a 2-layer neural network on top of feature representations for fine tuning. Two studies used saliency c-map for imaging feature visualization (34, 36). All four studies excluded patients with missing values in either imaging data or outcome measures. Three studies conducted k-fold cross-validation (34, 36, 37) and one used hold-out validation (35).

**Table 2:** Model development using deep learning algorithms.

**References**	**Outcomes**	**Missing value**	**Major imaging pre-processing**	**Model architecture**	**Feature visualization**	**Validation**
Hilbert et al. (34)	a. Good functional outcome (mRS ≤ 2) b. Successful reperfusion (TICI score≥2b)	Patients with missing data were excluded	a. Brain extraction (50–400 HU) b. Rigid registration to a template c. Computing maximum intensity projection from 3D to 2D scans d. Normalization e. Imaging resampling (368 × 432)	a. Functional outcome: supervised 2D-ResNet architecture with structured receptive field kernels model b. Successful reperfusion: a stacked denoising convolutional auto-encoder (2D-ResNet architecture with structured receptive field kernels) and fine-tuned model	Gradient-weighted Class Activation Mapping	4-fold cross validation
Samak et al. (35)	a. Good functional outcome (mRS ≤ 2) b. Individual mRS scores (0–6)	Patients with missing data were excluded	a. Brain extraction (40–100 HU) b. Data augmentation (flip, rotations, elastic deformations, Gaussian noise) c. Normalization d. Imaging resampling (192x192x32)	a. Multimodal model: image feature encoder, clinical metadata encoder, image and clinical metadata fusion b. 3D-convolutional kernels, attentional block	n.a.	Hold-out validation
Nishi et al. (36)	Good functional outcome (mRS ≤ 2)	Patients with missing data were excluded	a. Brain extraction b. Data augmentation (rotations, translation, spatial scaling) c. Normalization d. ROIs labeling (ischemic core lesion) e. Imaging resampling (128 × 128 × 32)	a. Multi-output model: A U-net segmentation task for imaging feature derivation, a 2-layer neural network for fine-tuning b. 3D-convolutional kernels	Gradient-weighted Class Activation Mapping	5-fold cross validation
Jiang et al. (37)	Hemorrhagic transformation (including HI1, HI2, PH1, and PH2)	Patients with missing data were excluded	a. Brain extraction b. Data augmentation (rotations, spatial scaling) c. ROIs labelling d. Imaging resampling (randomly cropped from ROIs)	a. Multimodal model: multiple imaging feature encoders (DWI, MTT, and TTP), clinical metadata encoder, image and clinical metadata fusion b. 3D-based convolutional kernels, Inception V3 architecture	n.a.	5-fold cross validation

mRS, Modified Rankin Scale; HI, hemorrhagic infarction; HU, Hounsfield Units; PH, parenchymatous hematoma; DWI, diffusion-weighted imaging; MTT, mean transit time; ROI: regions of interest; TTP, time to peak; TICI, thrombolysis in cerebral infarction score; n.a., not available.

### Model performance

#### Conventional machine learning algorithms

**Figure 2:**
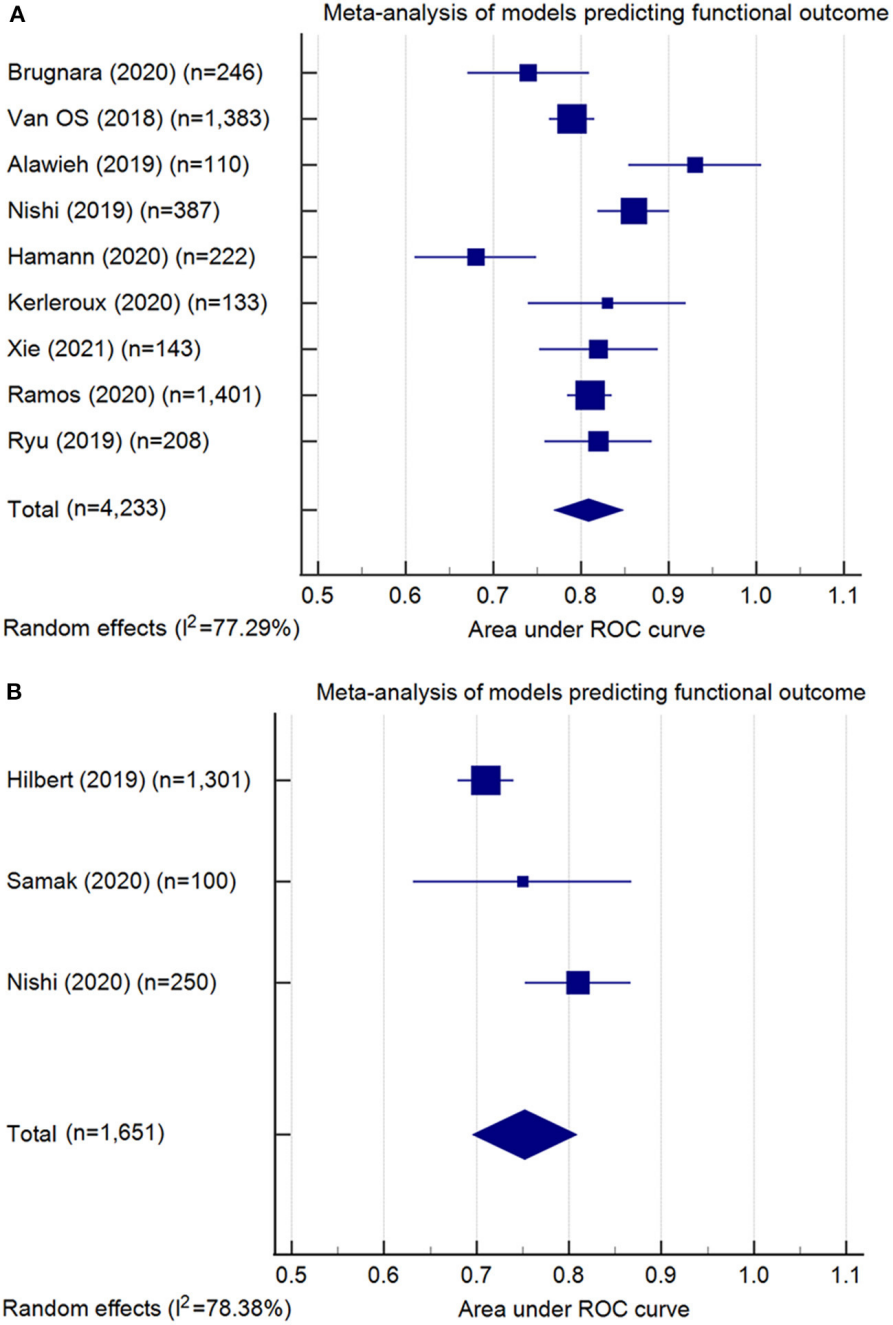
Meta-analysis of the area under the receiver-operating characteristics (ROC) curves (AUC) of models predicting functional outcome: **(A)** conventional machine learning models (pooled AUC = 0.81, 95% confidence interval: 0.77–0.85); **(B)** deep learning models (pooled AUC = 0.75, 95% confidence interval: 0.70–0.81). Note: Meta-analysis did not include the model developed by Kappelhof et al. (31), as the AUC was not reported.

Model performance of the 13 conventional ML models was summarized in Table 3. Ten models predicted the functional outcome at 90 days post-stroke defined by the mRS (39) (pooled AUC=0.81, 95% CI: 0.77–0.85, AUC range: 0.68–0.93, Figure 2A). Seven of these models used imaging features selected from computed tomography (CT) (pooled AUC=0.82, 95% CI: 0.78–0.86), and three involved features identified in magnetic resonance imaging (MRI) (pooled AUC=0.77, 95% CI: 0.70–0.85) (Supplementary Figure S1). Three models predicted successful reperfusion defined by the Thrombolysis in Cerebral Infarction Score (pooled AUC=0.72, 95% CI: 0.56–0.88, AUC range: 0.55–0.88; Supplementary Figure S2). Three models were validated (24, 25, 33) in external datasets.

**Table 3:** Model performance of conventional machine learning algorithms.

**Clinical outcome**	**Imaging modality**	**Clinical variable**	**Model**	**References**	**Sample size (T/EV)**	**Model performance**		**Validation**	
						**AUC (95% CI)**	**Others**	**Internal**	**External**
Good functional outcome at 90 days (mRS ≤ 2 or mRS ≤ 3)	NCCT and CTA	Yes	Gradient boosting decision trees	Brugnara et al. (22)	246	0.74 (0.73–0.75)	ACC,0.71	Yes	No
		Yes	RFA	Van et al. (23)	1,383^*^	0.79 (0.79–0.79)	n.a.	Yes	No
	NCCT	Yes	Regression trees	Alawieh et al. (24)	110/36	Internal: 0.93 (0.85–1.00)^†^ External: n.a.	Internal: n.a. External: PV+: 0.60, NV-: 0.95	Yes	Yes
		Yes	RLR	Nishi et al. (25)	387/115	Internal: 0.86 (0.78–0.94)^†^ External:0.90 (0.83–0.97)^†^	Internal: ACC,0.75; SEN,0.59; SPE,0.86; External: n.a.	Yes	Yes
	MRI (DWI and PWI)	Yes	RFA	Hamann et al. (26)	222	0.68 (0.61–0.76)	n.a.	Yes	No
		Yes	RFA	Kerleroux et al. (27)	133	0.83 (0.74–0.92)^†^	ACC,0.73; SEN,0.69; SPE,0.76	Yes	No
	MRI(DWI)	Yes	SVM	Xie et al. (28)	143	0.82 (0.75–0.89)^†^	ACC,0.77	Yes	No
Poor functional outcome at 90 days (mRS≥5 or mRS≥4)	NCCT and CTA	Yes	ANN	Ramos et al. (29)	1,401^*^	0.81 (0.79–0.83)	ACC, 0.65; SEN, 0.53; SPE,0.89; PV+, 0.69; NV-,0.80	Yes	No
	CTA	Yes	SVM	Ryu et al. (30)	482 (hold-out testing: 208)	0.82 (0.76–0.87)	n.a.	Yes	No
	n.a.	Yes	Decision trees	Kappelhof et al. (31)	1,090^*^	n.a	ACC,0.72	Yes	No
Successful reperfusion (TICI score≥2b)	NCCT and CTA	Yes	RFA	Van et al. (23)	1,383^*^	0.55 (0.55–0.56)	n.a.	Yes	No
Successful reperfusion at the first attempt (TICI score≥2b)	NCCT and CTA	No	RLR	Patel et al. (32)	119	0.77 (0.54–0.90)	ACC, 0.74	Yes	No
	NCCT and CTA	No	SVM	Hofmeister et al. (33)	109/47	External: 0.88 (0.75–1.00)^†^	External: ACC, 0.85; SEN, 0.50; SPE, 0.97, PV+, 0.86; NV-,0.85	Yes	Yes

ANN, artificial neural networks; AUC, area under the Receiver Operating Characteristic curve; ACC, accuracy; CTA, computed tomography angiography; DWI, diffusion weighted imaging; EV, external validation dataset; MRI, magnetic resonance imaging; NCCT, non-contrast computed tomography; NV-, negative predictive value; PWI, perfusion weighted imaging; PV+, positive predictive value; RFA, random forest analysis; RLR, regularized logistic regression; SVM, support vector machine; SEN, sensitivity; SPE, specificity; T, training dataset; n.a., not available/not applicable. Note:

* model derived from patients registered in MR CLEAN Registry (38).

† 95% CI was estimated based on normal distribution.

#### Deep learning algorithms

The six DL models were summarized in Table 4. Good functional outcome defined as mRS ≤ 2 was analyzed in three models (pooled AUC=0.75, 95% CI: 0.70–0.81; Figure 2B), among which two were CT-based (AUC range: 0.71–0.75) and one was MRI-based (AUC: internal, 0.81; external, 0.73). The outcomes predicted in the other three models include: each of the seven mRS points (accuracy=0.35), successful reperfusion (AUC=0.65, 95% CI: 0.62–0.68), and hemorrhage transformation (AUC=0.95, 95% CI: 0.87–1.00). Two models conducted external geographic validation (36, 37).

**Table 4:** Model performance of deep learning algorithms.

**Clinical outcome**	**Imaging modality**	**Clinical variable**	**Model**	**References**	**Sample size (T/EV)**	**Model performance**		**Validation**	
						**AUC (95% CI)**	**Others**	**Internal**	**External**
Good functional outcome at 90 days (mRS ≤ 2)	CTA	No	DL (RFNN)	Hilbert et al. (34)	1,301	0.71(0.68–0.74)^†^	n.a.	Yes	No
	NCCT	Yes	DL (CNN)	Samak et al. (35)	400 (hold-out testing: 100)	0.75 (0.63–0.87)^†^	ACC,0.77	Yes	No
	MRI (DWI)	No	DL (CNN)	Nishi et al. (36)	250/74	Internal: 0.81 (0.70–0.92)^†^ External:0.73 (0.61–0.85)^†^	Internal: SEN,0.76; SPE,0.76; ACC,0.72; External: SEN,0.72; SPE,0.60; ACC,0.65	Yes	Yes
Multiclass mRS (0, 1, 2, 3, 4, 5, 6) at 90 days	NCCT	Yes	DL (CNN)	Samak et al. (35)	400 (hold-out testing: 100)	n.a.	ACC, 0.35	Yes	No
Successful reperfusion (TICI score≥2b)	CTA	No	DL (RFNN)	Hilbert et al. (34)	1,301	0.65 (0.62–0.68)^†^	n.a.	Yes	No
Haemorrhagic transformation (including HI1, HI2, PH1, and PH2)	MRI (DWI and PWI)	Yes	DL (CNN)	Jiang et al. (37)	338/54	Internal: 0.95 (0.87–1.00)^†^ External:0.94 (0.85–1.00)^†^	Internal: SEN, 0.86; SPE, 0.90; ACC,0.89; External: SEN,0.86; SPE,0.89; ACC,0.88	Yes	Yes

ANN, artificial neural networks; AUC, area under the Receiver Operating Characteristic curve; ACC, accuracy; CTA, computed tomography angiography; CNN, convolutional neural network; DWI, diffusion weighted imaging; EV, external validation dataset; HI, hemorrhagic infarction; PH, parenchymatous hematoma; MRI, magnetic resonance imaging; NCCT, non-contrast computed tomography; NV-, negative predictive value; PWI, perfusion weighted imaging; PV+, positive predictive value; RFA, random forest analysis; RLR, regularized logistic regression; RFNN, receptive field neural networks; SVM, support vector machine; SEN, sensitivity; SPE, specificity; T, training dataset; n.a., not available/not applicable. Note: *model derived from patients registered in MR CLEAN Registry (38).^†^95% CI was estimated based on normal distribution.

### Risk of bias

Three ML-based studies (23, 29, 31) and one DL-based study (34) were considered at low risk of bias in all domains (Supplementary Table S5). The remaining studies were at high risk of bias in at least one domain (22, 24–28, 30, 32, 33, 35–37). Risk of bias mostly occurred in handling missing data. Risks of bias in other items, including standard outcome definition and internal validation techniques, was also identified.

### Reporting quality

All studies were rated as “good” in terms of overall adherence (>70% items reported) (Supplementary Table S6). However, several items remained rarely reported, including sample size calculations, how risk groups were defined, the detailed parameters of the prediction models and how to use the prediction model.

## Discussion

The application of ML techniques in prognostic prediction for LVO stroke is evolving. CT images have been more commonly used than MRI images in model development. Most studies used short-term reperfusion and functional outcomes at 90 days post-stroke as the prognostic endpoints. Conventional ML and DL models showed similar performance, but neither significantly outperformed existing prognostic scores. Also, many studies exhibited a high risk of potential bias and few studies adequately reported details of the models developed.

### Image data

Most studies selected CT over MRI as the imaging modality, in keeping with clinical practice (40). MRI may offer superior outcome prediction because of more precise measurement of early stroke damage, but its availability, acquisition speed and frequent contraindications have proven formidable barriers to routine use (41). Meanwhile, the performance of CT imaging has been improving over time, reducing the diagnostic precision gap (41). Indeed, our review suggests that MRI did not show superior performance to CT in prognostication, bolstering the rationale for developing CT-based prognostic models.

### Predicted outcomes

Our review identified clear gaps regarding the outcomes investigated. The only “long-term” outcome investigated was the mRS score at 90 days. This outcome was analyzed as a binary variable in all studies (dichotomized at two or three for good vs. moderate-to-poor outcome; or at four or five for poor vs. moderate-to-good outcome). However, such dichotomization might be arbitrary and inconsistent, which may have introduced a biased assessment of model performance if different thresholds were tested multiple times to obtain the “best” performance (19). Two studies (24, 35) also predicted each mRS point without dichotomizing the score, which may address a broader spectrum of functional status. On the other hand, a key outcome of clinical interest that remains un-investigated is futile recanalization, defined as poor functional outcomes at 90 days despite successful recanalization after EVT (42). Identification of those at high risk of futile recanalization is clinically and economically important, as an accurate prediction of this outcome would help avoid needless treatment and contribute to better resource allocation (42).

Accurate prediction of surrogate short-term outcomes may also help balance risk and benefit, and guide treatment approaches. There are two short-term outcomes investigated in the included studies—successful reperfusion and hemorrhagic transformation (HT). The model predicting HT (37) labeled all classes of HT as one category. However, it did not differentiate the symptomatic HT classes (i.e., PH2) from those classes without substantive mass effect (i.e., HT1 and HT2), and therefore may be of limited clinical utility. Also, there remains a gap in other relevant early outcomes. For example, occlusion at 24 h post-stroke, due to persistently failed recanalization or re-occlusion, has shown to be a predictor of longer-term outcomes in LVO patients (43) and may warrant investigation.

### Missing data

Missing data has been a general problem in medical datasets and was the most common potential cause of bias in the reviewed studies. Potential bias may be introduced when data are missing conditional on the observed data (44), so a systematic approach to dealing with missing data will improve the quality of a study, and hence should be considered. For a ML model, data may be missing in outcomes (labels), covariates, and medical images. For the former two, there is substantial knowledge regarding how to deal with missing data (45). Multiple imputation is generally recommended, as it leads to minimum bias by imputing missing values while preserving the original data characteristics (19, 44). In terms of missing imaging data, there are currently no generally accepted mitigatory methods, although this is an area of active methodological research (46).

### Model performance and limitations

Although conventional ML models can utilize a large quantity of clinical information, they have so far not demonstrated significant advantages against pre-treatment prognostic scores in predicting LVO outcomes (prognostic scores, AUC range: 0.61–0.80) (4). In contrast, a larger number of variables required in these models may limit the flexibility of their application in different clinical settings. Several conventional ML models achieved high performance values (AUCs: 0.86–0.93) (24, 25), but they were developed and validated in small datasets (sample size: development, 109–387; validation, 36–115) drawn from similar sampling frames (i.e., patients recruited in the same hospital at different time periods). ML models developed using small samples tend to be unstable and are likely to demonstrate substantially degraded predictive performance when applied to independent clinical populations (47). Overall, conventional ML models did not exhibit significant superiority when compared with prognostic scores for LVO outcome predictions.

Unlike conventional ML models that require variable selection, DL models are capable of analyzing raw imaging data in a “hypothesis-free” framework (9). However, the DL models in this review did not show superior performance to prognostic scores either. Most of these models were developed using small datasets, which may fail to capture the diverse features required to develop an accurate prognosis prediction model (48). This may also be one possible reason for the underwhelming performance. A few training schemes that suit clinical logics may help mitigate this issue (9). For example, augmenting data by mirroring CT images and inputting mirrored images with non-mirrored images enables the comparison between the affected side and the contralateral normal side, providing added information for model learning. Transfer learning from a clinically relevant task could also be a useful training scheme, e.g., pre-training the main task on an auxiliary task such as predicting occlusion of the left or right hemisphere. Further, multimodal data with richer information allows a model to capture diverse features and therefore may augment model performance. For example, multiple imaging modalities can provide diverse information, such as spatial information of hyperdense arteries, abnormal gray-white matter differentiation region and collateral supply (49). Similarly, non-imaging data can provide clinical-pathological features (i.e., blood glucose) that are associated with infarct progression and poor stroke outcomes (50). However, using multimodal imaging requires more computational resources, which may be a limiting factor for some research groups. Moreover, leveraging expert clinical knowledge is important to help augment model performance. For example, segmentation of hyperdense arteries or lesion and penumbra regions by experts allows additional information to be utilized in model development so that models can be trained to learn not only global features (i.e., location) but also fine details of the abnormal regions (i.e., boundary and shape).

### Barriers to real-world implementation

There are several barriers currently that may impede the clinical utilization of the models described in the current review. Firstly, only five models (26.3%) reviewed were validated externally. External validation in an out-of-distribution population tests the robustness and stability of model performance across different populations. For example, model performance may be impacted when the imaging data for model development have certain characteristics derived from different scanners and image acquisition protocols. Indeed, a study focusing on predicting retinopathy showed that the model performance degraded significantly when images were taken under poor lighting conditions and with lower imaging resolution (51). External validation can help verify that model performance is not impacted by unexpected factors and can identify models that are more generalizable to diverse populations of LVO patients—this is critical information for implementation in a local clinical setting (21, 52). Conversely, it is not sufficient to demonstrate performance without external validation (including prospective external validation) in a similar patient cohort. Over time, there are likely to be shifts in demographic composition and disease characteristics, as well as changes in new types of imaging scanners and image acquisition methods, even in the same center where that model was developed. A model tested only on an internal dataset may be brittle to these kinds of changes and see a drop in performance when used clinically. Secondly, only one study (32) published sufficient details of the models, including hyperparameters, coefficients (weights) and model equations, and only three studies (23, 26, 29) made the codes available online. Without the publication of sufficient details for independent model validation, it is difficult to directly implement published machine learning models in either validation studies or pre-clinical evaluation in local clinical environments. Current guidelines recommend the publication of “sufficient” details for validation, such as model structure, components, and values that used to control the learning process (hyperparameters) with code (19, 20, 53). For a deep learning model, it is difficult to publish millions of internal parameters in the paper, while it could be valuable to save files containing these parameters for future tasks as pre-trained weights. Thirdly, DL algorithms are usually described as “black box,” which may limit their explainability and acceptability for patients, clinicians, and policymakers (54). Visualization techniques such as saliency maps (55, 56) are used to aid in model interpretability in two included studies (34, 36) and do so by highlighting the regions of an image that contribute most to a classification decision. However, these techniques themselves require cautious interpretation as they can highlight portions of an image with both clinically relevant and irrelevant information, and an image can still be misclassified based on such information (54). Explainability techniques are prone to offer false reassurance that a model is behaving in an appropriate manner, and we should instead depend on thorough performance evaluation to engender trust in DL systems (54).

### Limitations and strengths

There are several limitations of our review. Firstly, we only included studies looking at LVO ischemic stroke treated with EVT treatment and did not examine studies including EVT for distal occlusion. However, as EVT is currently not a proven treatment for distal occlusion, any assessment of outcome prediction in this cohort is premature. Secondly, we have utilized re-calculated CIs for model comparisons and meta-analysis to ensure the similarity of the methods used. While most of the re-calculated 95% CIs are close to the original 95% CIs reported by included studies, we did note a significant deviation in the 95% CIs of two models (22, 32). We believe it is reasonable to rely on our wider estimate of CI compared to that provided in Brugnara et al. (22), as this original CI was extremely narrow based on a bootstrapping method and was much narrower than other 95% CIs reported on similar sized datasets. For the re-calculated CI that was narrower than the original report in Patel et al. (32), we again feel that the re-calculated version is more comparable to other studies as the small sample size resulted in less than 15 patients for the validation set, likely exaggerating the variability across cross-validation samples. This is the first comprehensive systematic review of ML and DL studies designed to predict clinical outcomes in LVO patients following EVT. Strengths of this review include a comprehensive literature search, independent screening and data extraction, as well as detailed quality assessment, all following PRISMA guidelines. More importantly, we conducted meta-analyses to quantitatively synthesize model performance, which has not been done in previous research that focused on ML and/or DL models for stroke prognostic prediction.

## Conclusions

ML and DL algorithms have been evolving rapidly and are being increasingly applied to prognostic prediction of LVO patients treated with EVT. However, the application of ML and DL to this field is at an early stage. The outcomes investigated so far are limited, and further studies may consider additional clinically important outcomes, such as futile recanalization and post-treatment complications. High risk of potential bias due to missing data and lack of reporting details of prediction models were seen in most studies. Following PROBAST and TRIPOD guidelines can help improve study quality and reporting transparency. The performance of conventional ML and DL models did not substantially differ from each other or from the performance of pre-existing simple prognostic scores. Although a few ML models achieved high performance, most were developed using small datasets and lacked solid external validation. There is potential for ML outcome prediction techniques to be superior to conventional techniques, though larger/diverse datasets, more rigorous data preprocessing, and solid external validation, are required before incorporation into clinical practice.

## Data availability statement

The original contributions presented in the study are included in the article/Supplementary material, further inquiries can be directed to the corresponding author.

## Author contributions

MZ contributed to study conception, design, collection and analysis of data, and draft writing. LJP contributed to the study design, data collection, data analysis, and critical revision of the manuscript. LOR contributed to the study design, data analysis, and critical revision of the manuscript. AB, LS, RS, TK, JJ, and MJ contributed to the study design and critical revision of the manuscript. ZW contributed to data collection and critical revision of the manuscript. All authors contributed to the article and approved the submitted version.

## Funding

MZ is supported by the Australian Government Research Training Program Scholarship. AB and LS are supported by the GlaxoSmithKline and the Australian Government Research Training Program Scholarship. LOR and LJP are supported by the GlaxoSmithKline. The funders were not involved in the study design, collection, analysis, interpretation of data, the writing of this article, or the decision to submit it for publication.

## Conflict of interest

The authors declare that the research was conducted in the absence of any commercial or financial relationships that could be construed as a potential conflict of interest.

## Publisher's note

All claims expressed in this article are solely those of the authors and do not necessarily represent those of their affiliated organizations, or those of the publisher, the editors and the reviewers. Any product that may be evaluated in this article, or claim that may be made by its manufacturer, is not guaranteed or endorsed by the publisher.
