# Supplementary material for: Pre-thrombectomy prognostic prediction of large-vessel ischemic stroke using machine learning: A systematic review and meta-analysis
Source: Front Neurol. 2022 Sep 8;13:945813. doi: 10.3389/fneur.2022.945813 (PMC9495610; doi:10.3389/fneur.2022.945813)

**Supplementary materials**

| **Table S1. Search strategies** | | |
| --- | --- | --- |
| Keywords | Search strategies | Result |
| **PubMed** |  |  |
| 1. Ischemic stroke and large vessel occlusion | ((((((("Brain Ischemia"[mh]) OR (brain ischemi* [tiab])) OR (brain ischaemi* [tiab])) OR ("Ischemic stroke"[mh])) OR (ischemic stroke [tiab])) OR (ischaemic stroke [tiab])) OR (cerebral ischaemi* [tiab])) OR (large vessel occlusion [tiab]) | 154,664 |
| 2. Artificial intelligence, convolutional neural network, random forests, Bayesian classifier, support vector machine, unsupervised learning, supervised learning, and natural language processing, machine learning, and deep learning | (((((((((("Artificial Intelligence"[mh]) OR (Artificial Intelligence[tiab])) OR (machine learning [tiab])) OR (deep learning [tiab])) OR (convolutional neural network* [tiab])) OR (random forest* [tiab])) OR (bayesian classifier [tiab])) OR (support vector machine* [tiab])) OR (unsupervised learning [tiab])) OR (supervised learning [tiab])) OR (Natural Language Processing [tiab]) | 202,852 |
| 1 and 2 | 1 and 2 | 739 |
| **Embase** |  |  |
| 1. Ischemic stroke and large vessel occlusion | exp 'brain ischemia'/ OR 'brain ischemi*':ti,ab,kw OR 'brain ischaemi*':ti,ab,kw OR 'ischemic stroke':ti,ab,kw OR 'ischaemic stroke':ti,ab,kw OR 'cerebral ischemi*':ti,ab,kw OR 'cerebral ischaemi*':ti,ab,kw OR 'large vessel occlusion':ti,ab,kw | 203,295 |
| 2. Artificial intelligence, convolutional neural network, random forests, Bayesian classifier, support vector machine, unsupervised learning, supervised learning, and natural language processing, machine learning, and deep learning | exp 'artificial intelligence'/ OR 'artificial intelligence':ti,ab,kw OR exp 'machine learning'/ OR 'machine learning':ti,ab,kw OR exp 'deep learning'/ OR 'deep learning':ti,ab,kw OR 'convolutional neural network*':ti,ab,kw OR 'random forest*':ti,ab,kw OR 'bayesian classifier':ti,ab,kw OR 'support vector machine*':ti,ab,kw OR 'unsupervised learning':ti,ab,kw OR 'supervised learning':ti,ab,kw OR 'natural language processing':ti,ab,kw | 311,304 |
| 1 and 2 | 1 and 2 | 1,049 |

| **Table S1. Search strategies (continues)** | | |
| --- | --- | --- |
| Keywords | Search strategies | Result |
| **Scopus** |  |  |
| 1. Ischemic stroke and large vessel occlusion | ( TITLE-ABS-KEY ( brain AND ischemia ) OR TITLE-ABS-KEY ( brain AND ischemi* ) OR TITLE-ABS-KEY ( brain AND ischaemi* ) OR TITLE-ABS-KEY ( ischemic AND stroke ) OR TITLE-ABS-KEY ( ischaemic AND stroke ) OR TITLE-ABS-KEY ( cerebral AND ischemi* ) OR TITLE-ABS-KEY ( cerebral AND ischaemi* ) OR TITLE-ABS-KEY ( large AND vessel AND occlusion ) ) | 261,282 |
| 2. Artificial intelligence, convolutional neural network, random forests, Bayesian classifier, support vector machine, unsupervised learning, supervised learning, and natural language processing, machine learning, and deep learning | ( TITLE-ABS-KEY ( artificial AND intelligence ) OR TITLE-ABS-KEY ( machine AND learning ) OR TITLE-ABS-KEY ( deep AND learning ) OR TITLE-ABS-KEY ( convolutional AND neural AND network* ) OR TITLE-ABS-KEY ( random AND forest* ) OR TITLE-ABS-KEY ( bayesian AND classifier ) OR TITLE-ABS-KEY ( support AND vector AND machine* ) OR TITLE-ABS-KEY ( unsupervised AND learning ) OR TITLE-ABS-KEY ( supervised AND learning ) OR TITLE-ABS-KEY ( natural AND language AND processing ) ) | 1,153,845 |
| 1 and 2 | ( ( TITLE-ABS-KEY ( artificial AND intelligence ) OR TITLE-ABS-KEY ( machine AND learning ) OR TITLE-ABS-KEY ( deep AND learning ) OR TITLE-ABS-KEY ( convolutional AND neural AND network* ) OR TITLE-ABS-KEY ( random AND forest* ) OR TITLE-ABS-KEY ( bayesian AND classifier ) OR TITLE-ABS-KEY ( support AND vector AND machine* ) OR TITLE-ABS-KEY ( unsupervised AND learning ) OR TITLE-ABS-KEY ( supervised AND learning ) OR TITLE-ABS-KEY ( natural AND language AND processing ) ) ) AND ( ( TITLE-ABS-KEY ( brain AND ischemia ) OR TITLE-ABS-KEY ( brain AND ischemi* ) OR TITLE-ABS-KEY ( brain AND ischaemi* ) OR TITLE-ABS-KEY ( ischemic AND stroke ) OR TITLE-ABS-KEY ( ischaemic AND stroke ) OR TITLE-ABS-KEY ( cerebral AND ischemi* ) OR TITLE-ABS-KEY ( cerebral AND ischaemi* ) OR TITLE-ABS-KEY ( large AND vessel AND occlusion ) ) ) | 1,345 |

| **Table S1. Search strategies (continues)** | | |
| --- | --- | --- |
| Keywords | Search strategies | Result |
| **Web of Science** |  |  |
| 1. Ischemic stroke and large vessel occlusion | TS = ((brain AND ischemia ) OR TITLE-ABS-KEY ( brain AND ischemi* ) OR ( brain AND ischaemi* ) OR ( ischemic AND stroke ) OR ( ischaemic AND stroke ) OR ( cerebral AND ischemi* ) OR ( cerebral AND ischaemi* ) OR ( large AND vessel AND occlusion )) | 208,966 |
| 2. Artificial intelligence, convolutional neural network, random forests, Bayesian classifier, support vector machine, unsupervised learning, supervised learning, and natural language processing, machine learning, and deep learning | TS = ((artificial AND intelligence ) OR ( machine AND learning ) OR ( deep AND learning ) OR ( convolutional AND neural AND network* ) OR ( random AND forest* ) OR ( bayesian AND classifier ) OR ( support AND vector AND machine* ) OR ( unsupervised AND learning ) OR ( supervised AND learning ) OR ( natural AND language AND processing ) ) | 636,841 |
| 1 and 2 | #1 AND #2 | 983 |

| **Table S2. PROBAST questions for risk-of-bias assessment** | |
| --- | --- |
| Participants | 1.1 Were appropriate data sources used, e.g., cohort, RCT, or nested case-control study data? |
|  | 1.2. Were all inclusions and exclusions of participants appropriate? |
| Predictors | 2.1. Were predictors defined and assessed in a similar way for all participants? (no assessment for deep learning models) |
|  | 2.2. Were predictor assessments made without knowledge of outcome data? (no assessment for deep learning models) |
|  | 2.3. Are all predictors available at the time the model is intended to be used? (no assessment for deep learning models) |
| Outcome | 3.1. Was the outcome determined appropriately? |
|  | 3.2. Was a prespecified or standard outcome definition used? |
|  | 3.3. Were predictors excluded from the outcome definition? (no assessment for deep learning models) |
|  | 3.4. Was the outcome defined and determined in a similar way for all participants? |
|  | 3.5. Was the outcome determined without knowledge of predictor information? (no assessment for deep learning models) |
|  | 3.6. Was the time interval between predictor assessment and outcome determination appropriate? |
| Analysis | 4.1 Were there a reasonable number of participants with the outcome? |
|  | 4.2. Were continuous and categorical predictors handled appropriately? (no assessment for deep learning models) |
|  | 4.3 Were all enrolled participants included in the analysis? |
|  | 4.4. Were participants with missing data handled appropriately? |
|  | 4.5. Was selection of predictors based on univariable analysis avoided? (no assessment for deep learning models) |
|  | 4.6. Were complexities in the data (e.g., censoring, competing risks, sampling of control participants) accounted for appropriately? |
|  | 4.7. Were relevant model performance measures evaluated appropriately? |
|  | 4.8. Were model overfitting, underfitting, and optimism in model performance accounted for? |
|  | 4.9. Do predictors and their assigned weights in the final model correspond to the results from the reported multivariable analysis? (no assessment for deep learning models) |

| **Table S3. TRIPOD checklist for reporting quality assessment** | |
| --- | --- |
| Title & Abstract | 1. Identify the study was development/validation/both, outcome of interest and mention machine/deep learning. |
|  | 2. Provide a summary of objectives, study design, setting, participants, sample size, predictors, outcome, statistical analysis, results, and conclusions. (no assessment regarding predictor aspect for deep learning models) |
| Background  &Objectives | 3a. Explain the medical context (including whether diagnostic or prognostic) and rationale for developing or validating the machine/deep model, including references to existing models. |
|  | 3b. Specify the objectives, including whether the study describes the development or validation of the model or both. |
| Methods | 4a. Describe the study design or source of data (e.g., randomized trial, cohort, or registry data), separately for the development and validation data sets, if applicable. |
|  | 4b. Specify the key study dates, including start of accrual; end of accrual; and, if applicable, end of follow-up. |
|  | 5a. Specify key elements of the study setting (e.g., primary care, secondary care, general population) including number and location of centres. |
|  | 5b. Describe eligibility criteria for participants. |
|  | 5c. Give details of treatments received, if relevant. |
|  | 6a. Clearly define the outcome that is predicted by the prediction model, including how and when assessed. |
|  | 6b. Whether any reporting on any actions to blind assessment of the outcome to other clinical data to be predicted. |
|  | 7a. Clearly define all predictors used in developing or validating the multivariable prediction model, including how and when they were measured (no assessment for deep learning models). |
|  | 7b. Report any actions to blind assessment of predictors for the outcome and other predictors (no assessment for deep learning models). |
|  | 8. Explain how the study size was arrived at. |
|  | 9. Describe how missing data were handled (e.g., complete-case analysis, single imputation, multiple imputation) with details of any imputation method. |
|  | 10a. Describe how predictors were handled in the analyses. (no assessment for deep learning models) |
|  | 10b. Specify type of model, all model-building procedures (including any predictor selection), and method for internal validation (no assessment regarding predictor aspect for deep learning models). |
|  | 10c. For validation, describe how the predictions were calculated. |
|  | 10d. Specify all measures used to assess model performance and, if relevant, to compare multiple models. |
|  | 10e. Describe any model updating (e.g., recalibration) arising from the validation, if done. |
|  | 11. Provide details on how risk groups were created, if done (no assessment for deep learning models). |
|  | 12. For validation, identify any differences from the development data in setting, eligibility criteria, outcome, and predictors. (no assessment regarding predictor aspect for deep learning models) |

| **Table S3. TRIPOD checklist for reporting quality assessment (continues)** | |
| --- | --- |
| Results | 13a. Describe the flow of participants through the study, including the number of participants with and without the outcome and, if applicable, a summary of the follow-up time. A diagram may be helpful. |
|  | 13b. Describe the characteristics of the participants (basic demographics, clinical features, available predictors), including the number of participants with missing data for predictors and outcome (no assessment regarding predictor aspect for deep learning models). |
|  | 13c. For validation, show a comparison with the development data of the distribution of important variables (demographics, predictors and outcome). (no assessment regarding predictor aspect for deep learning models) |
|  | 14a. Specify the number of participants and outcome events in each analysis. |
|  | 14b. If done, report the unadjusted association between each candidate predictor and outcome (no assessment for deep learning models). |
|  | 15a. Present the full prediction model to allow predictions for individuals (i.e., all regression coefficients, and model intercept or baseline survival at a given time point) (no assessment for deep learning models). |
|  | 15b. Explain how to use the prediction model (no assessment for deep learning models). |
|  | 16. Report performance measures (with CIs, p-value or range) for the prediction model. |
|  | 17. If done, report the results from any model updating (i.e., model specification, model performance). |
| Discussion | 18. Discuss any limitations of the study (such as nonrepresentative sample, few events per predictor, missing data) (no assessment regarding predictor aspect for deep learning models). |
|  | 19a. For validation, discuss the results with reference to performance in the development data, and any other validation data. |
|  | 19b. Give an overall interpretation of the results, considering objectives, limitations, results from similar studies, and other relevant evidence. |
|  | 20. Discuss the potential clinical use of the model and implications for future research. |
| Other information | 21. Provide information about the availability of supplementary resources, such as study protocol, Web calculator, and data sets. |
|  | 22. Give the source of funding and the role of the funders for the present study. |

| **Table S4. Basic characteristics of the eligible studies** | | | | | |
| --- | --- | --- | --- | --- | --- |
| Study | Age | Gender (male vs female) | Ethnicity/place of recruitment, periods of recruitment | Sample size (training dataset) | Occlusion sites |
| Brugnara (2020)(22) | Medians, 74.0-78.0 years | 48.0% vs 52.0% | Heidelberg, Germany; 2014-2018 | 246 | Anterior circulation occlusion |
| Van OS (2018)(23) | Mean, 69.8 years | 53.5% vs 46.5% | Netherlands national; 2014-2016 | 1383^*^ | Anterior circulation occlusion |
| Alawieh (2019)(24) | Means, training, 84.6 years  validation, 86.0 years | Training:  35.0% vs 65.0%  Validation: 46.0% vs 54.0% | Training: white vs not white, 63.0% vs 37.0%, 2013-2017  Validation: white vs not white, 69.0% vs 31.0%, 2017-2018 | 110 (external validation: 36) | Anterior or posterior circulation occlusion |
| Nishi (2019)(25) | Means: training, 76.0 years  validation, 77.4 years | Training:  53.3% vs 46.7%  Validation: 43.9% vs 56.1% | Training: Kobe and Kitakyushu, Japan; 2013-2018  Validation: Kyoto and Kobe, Japan; 2013-2018 | 387 (external validation: 115) | Anterior circulation occlusion |
| Hamann (2020)(26) | Median, 73.5 years | 40.0% vs 60.0% | Bern, Switzerland; 2012-2017 | 222 | MCA-M1 occlusion |
| Kerleroux (2021)(27) | Mean, 72.4 years | 65.9% vs 34.1% | France; 2015-2018 | 133 | Intracranial ICA or MCA-M1 occlusion |
| Xie (2021)(28) | Median, 64.0-68.0 years | 58.7% vs 41.3% | Nancy, France; 2009-2015 | 143 | Anterior circulation occlusion |
| Ramos (2020)(29) | Median, 71.0 years | 53.0% vs 47.0 % | Netherlands national; 2014-2016 | 1401^*^ | Anterior circulation occlusion |
| Ryu (2019)(30) | Means, 67.7-67.8 years | 45.0% vs 55.0% | Seoul, South Korea; 2010-2015 | 482 (hold-out testing: 208) | Anterior circulation occlusion |
| Kappelhof (2021) (31) | Median, 71.0 years | 53.0% vs 47.0% | Netherlands national; 2014-2016 | 1090^*^ | Anterior circulation occlusion |
| Patel (2021)(32) | Means, 70.5-75.7 years | 41.1% vs 58.9% | USA; 2018-2020 | 119 | MCA-M1 or M2 occlusion |
| Hofmeister (2020)(33) | Means, 73.9-74.7 years† | 53.2% vs 46.8%^†^ | Geneva, Switzerland; 2017-2018 | 109 (external validation: 47) | MCA-M1 or M2 occlusion |
| Hilbert (2019)(34) | Mean, 71.0 years | 53.4% vs 46.6% | Netherlands national; 2014-2016 | 1301^*^ | Anterior circulation occlusion |
| Samak (2020)(35) | Medians, 65.7-65.8 years | 58.4% vs 41.6% | Netherlands national; 2014-2016 | 400 (hold-out testing: 100)^*^ | Anterior circulation occlusion |
| Nishi (2020)(36) | Means, training, 74.4 years  validation, 75.7 years | Training: 58.4% vs 41.6%  Validation: 52.8% vs 47.2% | Training: Kobe and Kitakyushu, Japan; 2013-2018  Validation: Kyoto and Kobe, Japan; 2013-2018 | 250 (external validation: 74) | Anterior circulation occlusion |
| Jiang (2021)(37) | Means, 64.2-71.3 years | 62.2% vs 37.8% | Nanjing, China; 2016-2019 | 338 (external validation: 54) | n.a. |
| Abbreviations: MCA, middle cerebral artery; M1, horizontal segment of MCA; M2, insular segment of MCA; ICA, internal carotid artery. n.a., not applicable/available. Note: ^*^model derived from patients registered in MR CLEAN Registry(38). | | | | | |

**Table S5. Risk-of-bias assessment of each study by PROBAST questions**

| Study | 1.1 | 1.2 | 2.1 | 2.2 | 2.3 | 3.1 | 3.2 | 3.3 | 3.4 | 3.5 | 3.6 | 4.1 | 4.2 | 4.3 | 4.4 | 4.5 | 4.6 | 4.7 | 4.8 | 4.9 | Overall risk |
| --- | --- | --- | --- | --- | --- | --- | --- | --- | --- | --- | --- | --- | --- | --- | --- | --- | --- | --- | --- | --- | --- |
| Conventional machine learning algorithms | | | | | | | | | | | | | | | | | | | | |  |
| Brugnara (2020)(22) | PY | PY | PY | Y | Y | Y | PY | Y | Y | PY | Y | Y | Y | PY | PN | Y | Y | NI | Y | Y | + |
| Van OS (2018)(23) | Y | PY | Y | Y | Y | Y | PY | Y | PY | PY | Y | Y | Y | PY | Y | Y | Y | NI | Y | Y | - |
| Alawieh (2019)(24) | Y | Y | PY | Y | Y | Y | PY | Y | Y | PY | Y | Y | Y | PY | PN | Y | Y | NI | Y | Y | + |
| Nishi (2019)(25) | PY | PY | PY | Y | Y | Y | PY | Y | PY | PY | Y | Y | N | PY | PN | Y | Y | NI | Y | Y | + |
| Hamann (2020)(26) | Y | PY | PY | Y | Y | Y | PY | Y | Y | PY | Y | Y | Y | PN | PN | Y | Y | NI | Y | Y | + |
| Kerleroux (2021)(27) | PY | PY | PY | Y | Y | PY | PY | Y | Y | Y | Y | Y | NI | PY | PN | Y | Y | NI | Y | Y | + |
| Xie (2021)(28) | Y | PY | PY | Y | Y | Y | PY | Y | Y | Y | Y | Y | Y | PY | PN | Y | Y | NI | Y | Y | + |
| Ramos (2020)(29) | Y | PY | Y | Y | Y | Y | PY | Y | PY | PY | Y | Y | Y | PY | Y | Y | Y | NI | Y | Y | - |
| Ryu (2019)(30) | PY | PY | PY | Y | Y | Y | PN | Y | PY | PY | Y | Y | Y | PY | PN | Y | Y | NI | N | Y | + |
| Kappelhof (2021)(31) | Y | PY | Y | Y | Y | Y | PY | Y | PY | PY | Y | Y | Y | PY | Y | Y | Y | NI | Y | Y | - |
| Patel (2021)(32) | PY | PY | PY | Y | Y | Y | PY | Y | Y | PY | Y | Y | Y | PY | PN | Y | Y | NI | Y | Y | + |
| Hofmeister (2020)(33) | PY | PY | PY | Y | Y | Y | PY | Y | Y | PY | Y | N | Y | PY | PN | Y | Y | NI | Y | Y | + |
| Deep learning algorithms | | | | | | | | | | | | | | | | | | | | |  |
| Hilbert (2019)(34) | Y | PY | n.a. | n.a. | n.a. | Y | PY | n.a. | Y | n.a | Y | Y | n.a. | PY | PN | n.a. | Y | NI | Y | n.a. | -^*^ |
| Samak (2020)(35) | Y | PY | n.a. | n.a. | n.a. | Y | Y | n.a. | Y | n.a | Y | Y | n.a. | PY | PN | n.a. | Y | NI | N | n.a. | + |
| Nishi (2020)(36) | PY | PY | n.a. | n.a. | n.a. | Y | PY | n.a. | Y | n.a | Y | Y | n.a. | PN | PN | n.a. | Y | NI | Y | n.a. | + |
| Jiang (2021)(37) | PY | PY | n.a. | n.a. | n.a. | Y | Y | n.a. | PN | n.a | PY | Y | n.a. | PY | PN | n.a. | Y | NI | Y | n.a. | + |

Abbreviation: Y, yes for low risk of bias; PY, probably yes for low risk of bias; N, not for low risk of bias; PN, probably not for low risk of bias; NI, no information for risk of bias assessment; n.a., not assessed; +, indicated high risk of bias; -, indicated low risk of bias. ^*^Since multiple imputation is unlikely to be used in a deep learning study, we considered this study as low risk of bias although this study did not apply multiple imputation (item 4.4).

**Table S6. Quality assessment of each study by TRIPOD checklist**

| Study | 1 (D;V) | 2 (D;V) | 3a (D;V) | 3b (D;V) | 4a (D;V) | 4b (D;V) | 5a (D;V) | 5b (D;V) | 5c (D;V) | 6a (D;V) | 6b (D;V) | 7a (D;V) | 7b (D;V) | 8(D;V) | 9 (D;V) | 10 a (D) | 10b (D) | 10c (V) | 10d (D;V) | 10e (V) | 11 (D;V) | 12 (V) | 13a (D;V) | 13b (D;V) | 13c (V) | 14a (D) | 14b (D) | 15a (D) | 15b (D) | 16 (D;V) | 17 (V) | 18 (D;V) | 19a (V) | 19b (D;V) | 20 (D;V) | 21 (D;V) | 22 (D;V) |
| --- | --- | --- | --- | --- | --- | --- | --- | --- | --- | --- | --- | --- | --- | --- | --- | --- | --- | --- | --- | --- | --- | --- | --- | --- | --- | --- | --- | --- | --- | --- | --- | --- | --- | --- | --- | --- | --- |
| Conventional machine learning algorithms | | | | | | | | | | | | | | | | | | | | | | | | | | | | | | | | | | | | | |
| Brugnara (2020)(22) | Y | Y | Y | Y | Y | Y | Y | Y | Y | Y | Y | Y | Y | N | Y | Y | Y | n.a. | Y | n.a. | N | n.a. | Y | Y | n.a. | Y | Y | N | N | Y | n.a. | Y | n.a. | Y | Y | Y | Y |
| Van OS (2018)(23) | Y | Y | Y | Y | Y | Y | Y | Y | Y | Y | Y | Y | Y | N | Y | Y | Y | n.a. | Y | n.a. | N | n.a. | Y | Y | n.a. | Y | N | N | N | Y | n.a. | Y | n.a. | Y | Y | Y | Y |
| Alawieh (2019)(24) | Y | Y | Y | Y | Y | Y | Y | Y | Y | Y | Y | Y | Y | N | Y | Y | Y | Y | Y | N | N | Y | Y | Y | Y | Y | N | N | N | Y | N | Y | Y | Y | Y | Y | Y |
| Nishi (2019)(25) | Y | Y | Y | Y | Y | Y | Y | Y | Y | Y | Y | Y | Y | N | Y | Y | Y | Y | Y | N | N | Y | Y | Y | Y | Y | N | N | N | Y | N | Y | Y | Y | Y | Y | Y |
| Hamann (2020)(26) | Y | Y | Y | Y | Y | Y | Y | Y | Y | Y | Y | Y | Y | N | Y | Y | Y | n.a. | Y | n.a. | N | n.a. | Y | Y | n.a. | Y | Y | N | N | Y | n.a. | Y | n.a. | Y | Y | Y | Y |
| Kerleroux (2021)(27) | Y | Y | Y | Y | Y | Y | Y | Y | Y | Y | Y | Y | Y | N | Y | Y | Y | n.a. | Y | n.a | N | n.a | Y | Y | n.a | Y | Y | N | N | Y | n.a | Y | n.a | Y | Y | Y | Y |
| Xie (2021)(28) | Y | Y | Y | Y | Y | Y | Y | Y | Y | Y | Y | Y | Y | N | Y | Y | Y | n.a. | Y | n.a. | N | n.a. | Y | Y | n.a. | Y | Y | N | N | Y | n.a. | Y | n.a. | Y | Y | Y | Y |
| Ramos (2020)(29) | Y | Y | Y | Y | Y | Y | Y | Y | Y | Y | Y | Y | Y | N | Y | Y | Y | n.a. | Y | n.a. | N | n.a. | Y | Y | n.a. | Y | N | N | N | Y | n.a. | Y | n.a. | Y | Y | Y | Y |
| Ryu (2019)(30) | Y | Y | Y | Y | Y | Y | Y | Y | Y | Y | Y | Y | Y | N | Y | Y | Y | n.a. | Y | n.a. | N | n.a. | Y | Y | n.a. | Y | N | N | N | Y | n.a. | Y | n.a. | Y | Y | Y | Y |
| Kappelhof (2021)(31) | Y | Y | Y | Y | Y | Y | Y | Y | Y | Y | Y | Y | Y | N | Y | Y | Y | n.a. | Y | n.a. | N | n.a. | Y | Y | n.a. | Y | N | N | N | N | n.a. | Y | n.a. | Y | Y | Y | Y |
| Patel (2021)(32) | Y | Y | Y | Y | Y | Y | Y | Y | Y | Y | Y | Y | Y | N | Y | Y | Y | n.a. | Y | n.a. | N | n.a. | Y | Y | n.a. | Y | Y | Y | Y | Y | n.a. | Y | n.a. | Y | Y | Y | Y |
| Hofmeister (2020)(33) | Y | Y | Y | Y | Y | Y | Y | Y | Y | Y | Y | Y | Y | N | Y | Y | Y | Y | Y | N | N | Y | Y | Y | N | Y | Y | N | N | Y | N | Y | Y | Y | Y | Y | Y |
| Deep learning algorithms | | | | | | | | | | | | | | | | | | | | | | | | | | | | | | | | | | | | | |
| Hilbert (2019)(34) | Y | Y | Y | Y | Y | Y | Y | Y | Y | Y | Y | n.a. | n.a. | N | Y | n.a. | Y | n.a. | Y | n.a. | n.a. | n.a. | Y | Y | n.a. | Y | n.a. | n.a. | n.a. | Y | n.a. | Y | n.a. | Y | Y | Y | Y |
| Samak (2020)(35) | Y | N | Y | Y | Y | Y | Y | Y | Y | Y | Y | n.a. | n.a. | N | Y | n.a. | Y | n.a. | Y | n.a. | n.a. | n.a. | Y | Y | n.a. | Y | n.a. | n.a. | n.a. | N | n.a. | Y | n.a. | Y | Y | Y | Y |
| Nishi (2020)(36) | Y | Y | Y | Y | Y | Y | Y | Y | Y | Y | Y | n.a. | n.a. | N | Y | n.a. | Y | Y | Y | N | n.a. | Y | Y | Y | Y | Y | n.a. | n.a. | n.a. | Y | N | Y | Y | Y | Y | Y | Y |
| Jiang (2021)(37) | Y | Y | Y | Y | Y | Y | Y | Y | Y | Y | Y | n.a. | n.a. | N | Y | n.a. | Y | Y | Y | N | n.a. | Y | Y | Y | Y | Y | n.a. | n.a. | n.a. | Y | N | Y | Y | Y | Y | Y | Y |
| Abbreviation: Y, reported; N, didn’t report; n.a., not assessed. D, items relevant to the development of a prediction model; V, items relevant to the validation of a prediction model; D;V, items relevant to both of the development and validation of a prediction model. | | | | | | | | | | | | | | | | | | | | | | | | | | | | | | | | | | | | | |

**Figure S1. Meta-analysis of the area under the receiver-operating characteristics (ROC) curves (AUC) of models predicting functional outcome: (A) CT-based models (pooled AUC = 0.82, 95% confidence interval: 0.78-0.86); (B) MRI-based models (pooled AUC = 0.77, 95% confidence interval: 0.70-0.85)**

Note: Meta-analysis did not include the model developed by Kappelhof(31), as the AUC was not reported.


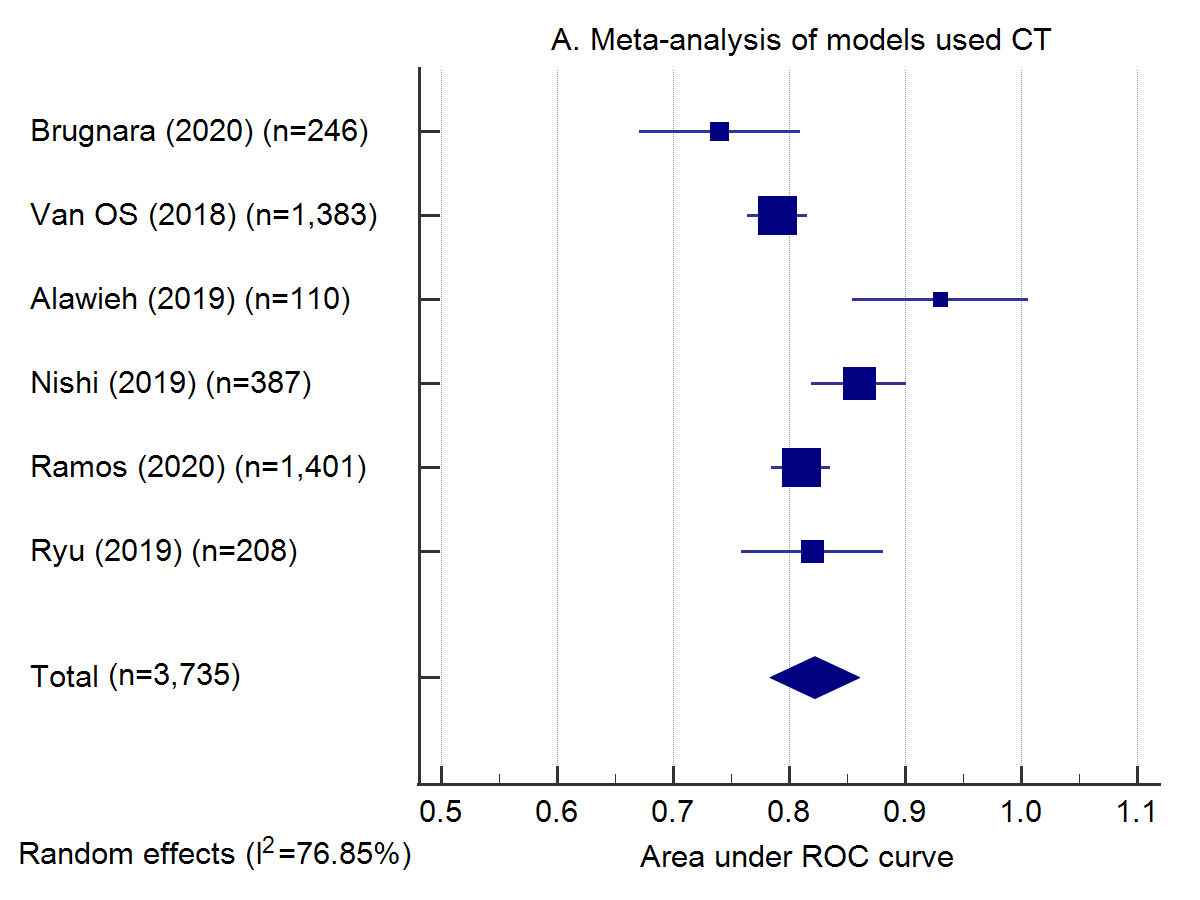


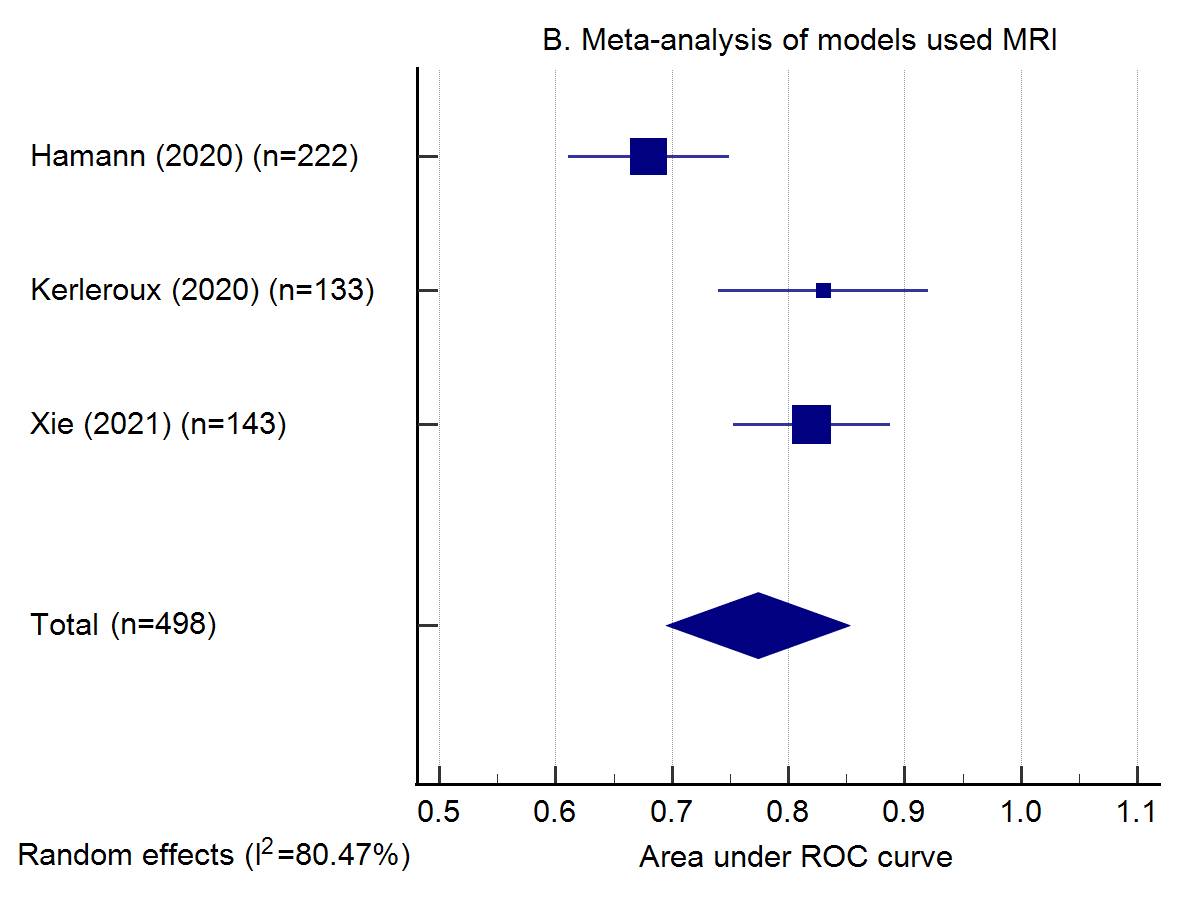


**Figure S2. Meta-analysis of the area under the receiver-operating characteristics (ROC) curves (AUC) of models predicting successful reperfusion** (pooled AUC = 0.72, 95% confidence interval: 0.56-0.88)

Note: There is only one deep learning model(34) that predicted successful reperfusion, so meta-analysis was not performed for this outcome.


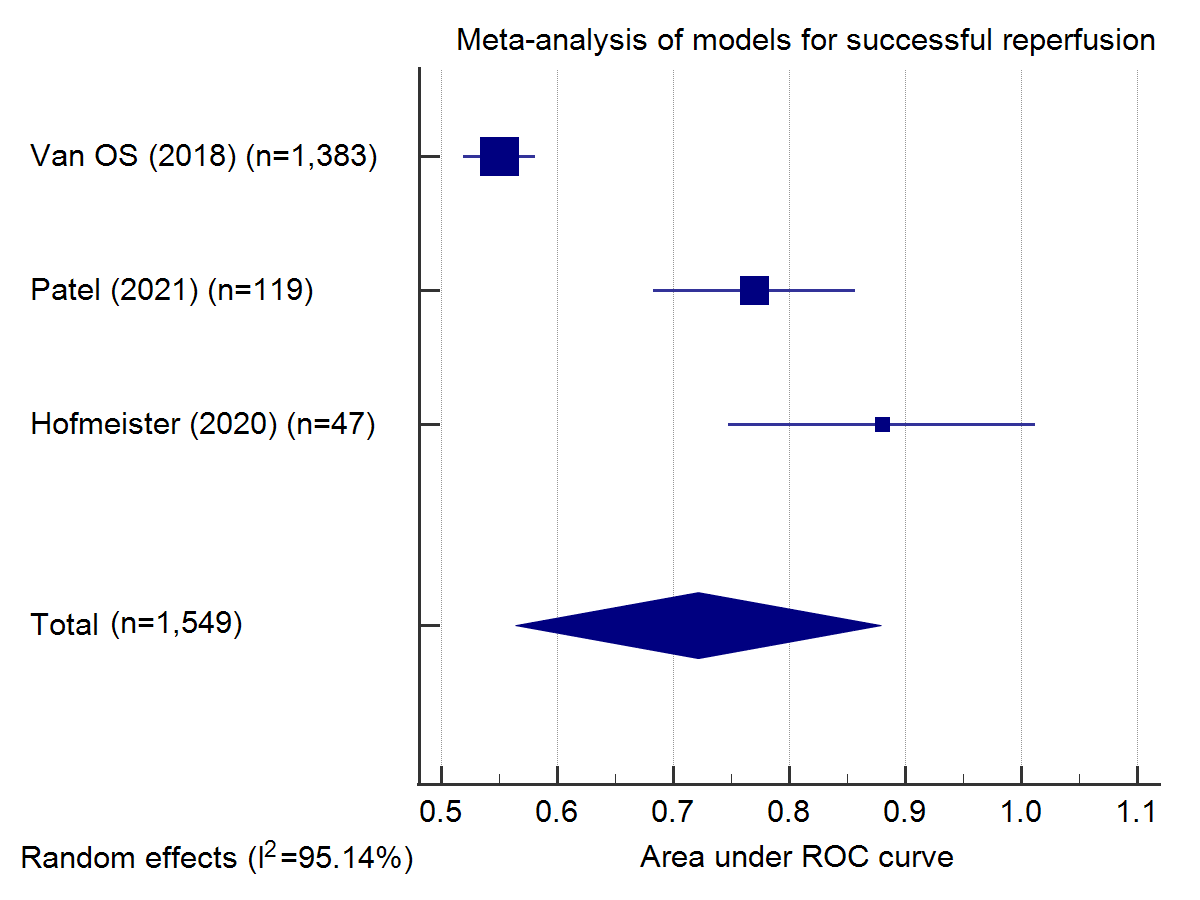

Supplement: Supplementary file 1 [file Data_Sheet_1.docx]
